# Optical puncture combined with balloon dilation PCNL vs. conventional puncture dilation PCNL for kidney stones without hydronephrosis: a retrospective study

**DOI:** 10.1186/s12894-019-0558-1

**Published:** 2019-11-27

**Authors:** Mi Zhou, Xiang He, Yuelong Zhang, Weiwen Yu

**Affiliations:** Department of Urology, Zhejiang Provincial People’s Hospital/People’s Hospital of Hangzhou Medical College, Hangzhou, China

**Keywords:** Optical puncture, Balloon dilation, Percutaneous nephrolithotomy, Kidney stones without hydronephrosis

## Abstract

**Background:**

Accurate puncture and dilation of the target kidney calices for percutaneous nephrolithotomy (PCNL) can be difficult. This study aimed to investigate the advantages of PCNL using optical puncture (i.e. the puncture is visualized on a screen as seen through the needle) combined with balloon dilation vs. conventional puncture methods.

**Methods:**

This was a retrospective study of 58 consecutive patients with kidney stones without hydronephrosis and treated at the Minimally Invasive Urology Center of Zhejiang Provincial People’s Hospital between 10/2016 and 12/2017. Twenty-one patients underwent optical puncture combined with balloon dilation PCNL. Thirty-seven patients underwent conventional puncture instrument dilation PCNL (controls). Success rate, tubeless rate, blood loss, pain, and complications were compared between the two groups.

**Results:**

The one-time puncture success rate (95.2% [20/21] vs. 67.6% [25/37], *P* = 0.02) and the postoperative tubeless rate (81.0% [17/21] vs. 54.1% [20/37], *P* = 0.04) were higher in the optical puncture group compared with controls. The average postoperative hemoglobin reduction was smaller (1.13 ± 0.63 vs. 1.56 ± 0.59 g/dL, *P* = 0.01), the postoperative VAS score was lower (1.6 ± 0.9 vs. 2.5 ± 1.2, *P* = 0.004), the rate of postoperative analgesic use was lower (14.3% [3/21] vs. 40.5% [15/37], *P* = 0.04), and the postoperative mean hospitalization days was shorter (3.7 ± 0.9 vs. 4.4 ± 0.8, *P* = 0.005) in the optical puncture group vs. controls. There was no case of urinary sepsis, blood transfusion, perirenal hematoma, pleural injury, and visceral organ damage.

**Conclusions:**

Optical puncture combined with balloon dilation PCNL could be associated with good therapeutic effect and low frequency of complications for the treatment of kidney stones without hydronephrosis.

## Background

Most kidney stones do not lead to significant symptoms, but partial, intermittent, or complete urinary tract obstruction will result in pain [1]. The reported lifetime incidence of kidney stone is 1–15%, depending on countries and lifestyle habits [2, 3], and the incidence is high in Western countries and in children [4, 5]. In China, the prevalence of kidney stones is around 7.5% and shows a rising trend [6].

Percutaneous nephrolithotomy (PCNL) is the main minimally invasive surgical method for the treatment of large or complex stones [7]. The successful establishment of the percutaneous renal channel is the basis for PCNL. Accurate puncture and dilation of the target kidney calices are keys to establish the percutaneous renal channel. The renal cortex in patients with kidney stones without hydronephrosis is relatively thick, the space in the target kidney calices is limited, and routine PCNL puncture and dilation are associated with risks of hemorrhage and channel loss [8]. Therefore, the selection of the best PCNL approach for the treatment of kidney stones without hydronephrosis is still a challenge for urologists.

Optical puncture technology can be used to confirm whether the puncture site is reliable and reasonable [9, 10]. The application of balloon dilation can reduce the damage to the renal parenchyma [11–13]. The combination of the two could decrease the risk of complications of kidney stones without hydronephrosis and improve the postoperative stone clearance rate.

Therefore, this study was designed to investigate the advantages of optical puncture combined with balloon dilation PCNL. Although observational, this study could provide some basis for the selection of the best PCNL approach and for an eventual randomized controlled trial.

## Methods

### Study design and patients

This was a retrospective analysis of 58 consecutive patients with kidney stones without hydronephrosis and treated at the Minimally Invasive Urology Center of Zhejiang Provincial People’s Hospital between October 1, 2016 and December 31, 2017. The study was approved by the ethics committee of Zhejiang Provincial People’s Hospital. The need for individual consent was waived by the committee because of the retrospective nature of the study.

The inclusion criteria were: 1) kidney stones maximum diameter of > 2 cm; and 2) no hydronephrosis or mild hydronephrosis (level 1 or 2 in Grignon’s grading) by IVP/CTU examination [14]. The exclusion criteria were: 1) distal stone obstruction; 2) severe heart or lung disease; 3) diseases with hemorrhagic tendency; 4) ASA grade III or above; 5) solitary kidney or functional solitary kidney; 6) abnormal renal function; or 7) upper urinary tract infection with unsatisfying preoperative control.

### Preoperative examinations

All patients completed the preoperative examinations including blood routine, blood type, urine routine, urine culture, stool routine, occult blood, biochemical tests (liver and kidney function, fasting blood glucose, lipids, and electrolytes), coagulation function, thyroid function, parathyroid function, tests for infectious diseases (hepatitis B, hepatitis C, syphilis, and AIDS, as per Chinese guidelines for PCNL), electrocardiogram, chest X-ray, abdominal ultrasound, urinary B ultrasound, and IVP/CTU.

### Surgical methods

All procedures were conducted by three senior physicians (> 20 years of experience and completed more than 500 PCNL procedures) under general anesthesia. After effective anesthesia, a F6 ureteral catheter was implanted into the ureters in the lithotomy position (to prevent the stone fragment moving to the distal ureter during operation). A 16F Foley urethral catheter was implanted into the bladder. Then, the patient was converted to the prone position. Puncture was performed under ultrasound positioning. The puncture site was between the scapular line and the posterior axillary line under the 11th or 12th rib. The puncture entered into the renal calyx at the fornix.

For optical puncture combined with balloon dilation PCNL, a 16 G (1.5 mm) Kangge puncture needle (Shanghai Shangyi Kangge Medical Equipment Co., Ltd., China) was used for puncture after B-mode ultrasound (BK Ultrasound Machines, Denmark) positioning. Two optical methods were used during the study period. The UMP nephroscope (F3, Schoelly, Germany) was used to monitor the whole puncture process through the lumen of the puncture needle by the visualization platform; or the process was observed with a Poly optical puncture nephroscope (F4.8, Poly, Germany) until the puncture needle entered the target renal calyx and the stones were seen. The visualization approach, the type of puncture instrument, the type of guide wire, and the brand of the dilation balloon were selected according to the physicians’ experience.

A Bard (Bard Biopsy Systems, Tempe, AZ, USA)/Cook (Cook Medical, Bloomington, IN, USA) super smooth guide wire was placed through a puncture needle and a Bard (Bard, USA)/Cook (Cook, USA) nephroscope dilation balloon catheter was placed along the guide wire. B-mode ultrasound positioning was used to confirm that the balloon was in the target renal calyx. Saline was injected into the balloon and the water pressure was set to 20–25 atm for coaxial radial expansion and maintained for 3–5 min. A 24 Fr outer sheath was placed along the balloon catheter into the target renal calyx. The balloon catheter was withdrawn. A 18 Fr Wolf nephroscope (Richard-wolf, Germany) was used to start the lithotripsy procedure. The perfusion pump was used to infuse physiological saline into the collecting system during lithotripsy and flow rate was kept at 350–400 ml per minute. Ultrasound combined with a pneumatic ballistic suction system (EMS, Switzerland) was used for lithotripsy and clearing the stones. After lithotripsy, the collecting system was carefully examined for each renal calyx and the junctions of the renal pelvis and ureter. B-mode ultrasound was used for confirmation of no apparent residual stones left. A super smooth guide wire was placed into the collecting system. The F6 ureteral catheter was removed and a 6 Fr DJ stent was indwelled into the ureter along the guide wire. The puncture channel and renal parenchyma were observed for bleeding. A 20 Fr nephrostomy tube was indwelled. The 24 Fr outer sheath was gradually withdrawn. Dressing was directly applied on the skin incision.

If hemorrhage was found at the end of operation, a bipolar columnar electrocoagulation rod/Nd laser was used to stop the hemorrhage along the channel. If there were no injury to the collecting system, no severe hydronephrosis, no intrarenal infection, and no residual stone > 4 mm, the nephrostomy tube was not indwelled (postoperative tubeless). If there were no visible bleeding and residual stones after surgery, the DJ stent was not indwelled (completely postoperative tubeless).

In the control group, the conventional punctures were performed using the Create puncture instrument (Dalian Create Medical Products Co., Ltd., China) or the UROVISION puncture instrument (LAKH, Inc., Germany). Puncture needles (18G) were used to puncture the target renal calyx under ultrasound guidance. After successful puncture, a Bard (Bard, USA)/Cook (Cook, USA) super smooth guide wire was implanted. After the skin was incised, the percutaneous renal passage was dilated to 24 Fr using a fascial dilator and another sheath was indwelled. The remaining operations were similar to the operations of optical puncture combined with balloon dilation PCNL, as above.

### Postoperative management

Blood routine, CRP, PCT, and coagulation function examinations were completed on the day of surgery and the following day. Postoperative blood loss, inflammatory responses, and stability of the internal environment were evaluated. Antibiotics were routinely used for 3–5 days after surgery. The patients’ vital signs, urine color, and urine volume were observed. CT plain scan of the urinary system was repeated 1–3 days after surgery to assess whether there were any perinephric effusion and hematocele after surgery, complications, residual stones, and whether the nephrostomy tube should be removed. All patients were given non-steroidal anti-inflammatory drugs on the day of surgery. Postoperative pain was evaluated by visual analogue scale (VAS) on the following day. If VAS was ≥3, the patients continued to use analgesics. The patient was required to stand and be active 1–3 days after surgery and adjust the amount of activity according to the change of urine color. Normal diet was given after postoperative defecation. If there was obviously active hematuria after surgery, digital subtraction angiography (DSA) hemostasis was completed as soon as possible. The urethral catheters and nephrostomy tubes were removed 2–7 days after surgery (for patients with nephrostomy tube) and the patients were discharged. Stone specimens were physicochemically analyzed by infrared spectroscopy. DJ stents were removed at the outpatient clinic one month after surgery (for patients with DJ stents). CT plain scan of the urinary system was performed to evaluate the stone-free rate (SFR), defined as no residual stones or residual stones with a maximum diameter ≤ 4 mm and no clinical symptoms [15]. If the stone-free state was not achieved, CT plain scan of the urinary system was performed again 3 months after surgery to assess the SFR.

### Follow-up

Since this study aimed to observe the surgical complications and the postoperative SFR, only 3 months of follow-up after surgery was considered.

### Statistical analysis

SPSS 19.0 (IBM, Armonk, NY, USA) was used for statistical analysis. Continuous data were tested for normal distribution using the Kolmogorov-Smirnov test. All data were found to be normally distributed, presented as means ± standard deviation, and analyzed using the Student t test. Categorical data were presented as frequencies and analyzed using the Fisher’s exact test. Two-sided *P* values ≤0.05 were considered statistically significant.

## Results

### Characteristics of the patients

General clinical data and postoperative analysis of stone composition in the 21 patients in the optical puncture group and the 37 patients in the control group are shown in Table 1. There were 35 males and 23 females. They were 21–77 years of age (mean of 45.2 ± 13.4 years). The mean maximum diameter of the stones was 3.9 ± 1.2 cm. There were 16 patients with single stones and 42 with multiple stones (25 patients with stag horns). The kidney stones were on the left side in 22 patients and on the right side in 36. The ASA scores were I in 34 patients and II in 24. Thirteen patients had diabetes mellitus, 19 had hypertension, and three had coronary heart disease. There were no significant differences in sex, age, ASA score, stone size, location, number of stones, and stone composition between the two groups.

**Table 1:** Characteristics of the patients and stones

	Optical puncture group (*n* = 21)	Control group (*n* = 37)	P
Sex, n (%)			0.710
Male	12 (57.1)	23 (62.2)	
Female	9 (42.9)	14 (37.8)	
Mean age (years)	46.8 ± 12.4	44.2 ± 14.0	0.483
Maximum stone diameter (mm)	4.0 ± 1.0	3.9 ± 1.3	0.666
Number of stones, n (%)			0.900
Single	6 (28.6)	10 (27.0)	
Multiple	15 (71.4)	27 (73.0)	
Stone position, n (%)			0.985
Left side	8 (38.1)	14 (37.8)	
Right side	13 (61.9)	23 (62.2)	
ASA score, n (%)			0.431
I	12 (57.1)	25 (67.6)	
II	9 (42.9)	12 (32.4)	
Stone composition, n (%)			0.822
Calcium oxalate	3 (14.3)	5 (13.5)	
Calcium phosphate	2 (9.5)	6 (16.2)	
Magnesium phosphate	3 (14.3)	4 (10.8)	
Uric acid	1 (4.8)	1 (2.7)	
Mixed	12 (57.1)	21 (56.8)	

### Surgery and therapeutic effect

Surgical characteristics and therapeutic effects are shown in Table 2. The first-time puncture success rate (95.2% vs. 67.6%, *P* = 0.02) and the postoperative tubeless rate (81.0% vs. 54.1%, *P* = 0.04) were higher in the optical puncture group compared with controls. Postoperative hemoglobin reduction was lower (1.13 ± 0.63 vs. 1.56 ± 0.59 g/dL, *P* = 0.01), postoperative VAS score was lower (1.6 ± 0.9 vs. 2.5 ± 1.2, *P* = 0.004), rate of postoperative analgesic use was lower (14.3% vs. 40.5%, P = 0.04), and mean postoperative hospitalization days was shorter (3.7 ± 0.9 vs. 4.4 ± 0.8, *P* = 0.005) in the optical puncture group compared with controls. There were no significant differences in the mean operation time and completely postoperative tubeless rate. Urethral catheters and nephrostomy tubes (for patients with nephrostomy tubes) were successfully removed for all patients before discharge. The DJ stents were removed one month after surgery (for patients with DJ stents).

**Table 2:** Surgical data and therapeutic effect

	Optical puncture group (*n* = 21)	Control group (*n* = 37)	P
Mean operation time (min)	57 ± 8	53 ± 7	0.058
One-time success rate of puncture, n (%)	20 (95.2)	25 (67.6)	0.016
Hemorrhage			
Mean reduction of postoperative Hb (g/dL)	1.13 ± 0.63	1.56 ± 0.59	0.012
Postoperative DSA rate, n (%)	1 (4.8)	2 (5.4)	0.916
Infection, n (%)			
Postoperative fever rate	2 (9.5)	4 (10.8)	0.878
Incidence of urinary sepsis	0	0	1
Pain situation			
Postoperative VAS score	1.6 ± 0.9	2.5 ± 1.2	0.004
Postoperative analgesic usage, n (%)	3 (14.3)	15 (40.5)	0.039
Average postoperative hospitalization days	3.7 ± 0.9	4.4 ± 0.8	0.005
Postoperative tubeless rate, n (%)	17 (81.0)	20 (54.1)	0.042
Postoperative completely tubeless rate	23.8%(5)	18.9%(7)	0.661
Stone-free rate after 1 month of operation, n (%)	16 (76.2)	29 (78.3)	0.849
Stone-free rate after 3 month of operation, n (%)	20 (95.2)	35 (94.6)	0.916

### Complications

There were two and four patients with postoperative fever in the optical puncture and control groups, respectively. All of them were transient fever, without clinical manifestations of urinary sepsis. Fresh gross hematuria was observed in two and five patients, respectively. Among them, one and two patients, respectively, had DSA embolization for hemostasis. No patients required blood transfusion. The postoperative CT examination showed no significant perirenal hematoma, no pleural injury, or visceral organ damage.

### Residual stones

In the optical puncture group, residual stones were found in five patients who underwent CT scan reexamination at 1 month after surgery. There was still one patient with residual stones at 3 months after surgery, which was treated with extracorporeal lithotripsy. In the control group, residual stones were found in eight patients who underwent CT scan reexamination at 1 month after surgery. There were still two patients with residual stones at 3 months after surgery. One patient underwent extracorporeal lithotripsy and the other one underwent ureteroscopic lithotripsy. All remaining patients achieved SFR on CT scan at 3 months after surgery.

## Discussion

PCNL is difficult because the space of the target calyces is small. Therefore, this study was designed to investigate the advantages of optical puncture combined with balloon dilation PCNL. The results suggest that optical puncture combined with balloon dilation PCNL could be associated with good therapeutic effect and small frequency complications for the treatment of kidney stones without hydronephrosis.

In 1976, Fernström et al. [16] first reported the PCNL minimally invasive method for the treatment of urolithiasis. At present, PCNL is still the first choice for the treatment of kidney stones and upper ureteral stone of > 2 cm or > 500 mm^2^ [17, 18]. According to the size of the channel, percutaneous nephroscopes can be divided into standard-channel (24F–30F) or conventional-channel (>22F), mini-channel (14-22F), ultra/super-mini-channel (12F–14F), mini-micro-channel (8F), and micro-channel (<5F) [19–22]. The preferred channel for treating stag horn stone is still standard-channel PCNL [23]. In the treatment of large stones, negative pressure suction combined with standard-channel puncture and using ultrasound and pneumatic lithotripsy could effectively shorten the operation time, reduce intrarenal perfusion pressure during surgery, increase stone clearance rate, and reduce complications during and after the operation, especially the occurrence of urinary sepsis [24, 25]. Nevertheless, surgery with standard channel may increase the risk of bleeding. It requires accurate puncture techniques and effective channel establishment. In cases of kidney stone without hydronephrosis, the ultrasound localization of kidney stone is limited, and it is easy to lose the channel when the channel is established because the effective space of the renal calyces is small, increasing the difficulty of operation.

In 1997, Bellman et al. [26] selected 50 patients who underwent tubeless PCNL surgery and retained only DJ stent after operation, and compared them with 50 cases of conventional catheterization. They found that the tubeless treatment group had a lower use of analgesics, shorter hospitalization, and reduced treatment costs compared with the traditional catheterization group. In a prospective trial, Aghamir et al. [27] randomized 70 patients to a completely tubeless technique (no retention of nephrostomy tube and DJ stent) and a traditional catheterization group, and the outcomes were similar (i.e. there were no significant differences in postoperative bleeding, postoperative complications, secondary operation rate, and stone removal rate). The premise of tubeless PCNL is that kidney bleeding is controlled at the end of the operation and kidney drainage is unobstructed after operation. Therefore, the tubeless PCNL requires more precise surgical techniques, but there should be no bleeding, no iatrogenic injury, and no residual stone at the end of operation.

In patients with kidney stone without hydronephrosis, the traditional method aims to establish artificial hydronephrosis in order to increase the success rate of targeted calyceal puncture [28]. A ureteral catheter placed on the affected side prior to puncture, and the assistant pushes saline into the targeted renal calyx, which increases the success rate of puncture. But sometimes water injection will increase the intrarenal pressure of the renal pelvis and renal calyx, and it may increase the risk of septicemia. Recently, studies reported an improved success rate of renal puncture using GPS ultrasound positioning systems [29, 30]. Indeed, the GPS ultrasound positioning system can be used to adjust the angle and direction of the puncture needle in real time, and guide the depth of puncture. It could reduce the difficulty of puncture to a certain extent. It is still based on ultrasound and had no advantage to determine the calyceal fornix without hydronephrosis. In addition, its real-time location depended on the magnetic field sensing system, and errors may occur. Jie et al. [31] used B-mode ultrasound combined with X-ray to guide the puncture, and reported improved accuracy of puncture by using the two methods. Nevertheless, this combined method requires the repeated use of B-mode ultrasound and C-arm X-ray, which lengthen the operation time. In addition, the repeated use of the C-arm X-ray machine increases radiation exposure of patients and medical personnel.

In the present study, the optical puncture technique was used to improve the success rate of the puncture of kidney without hydronephrosis. X-ray is not required in this method. The optical puncture technique was first reported by Bader et al. [9]. Using this method, the whole puncture process is completed under direct vision for entering into the target renal calyx. Desai et al. [10] and Hanqing et al. [10, 32] used the optical puncture system to perform ultra-microchannel lithotripsy to treat kidney stones < 2 cm, and achieved good surgical results. In the present study, seven patients were treated with a 16G (1.5 mm) Kangge puncture needle combined with UMP nephroscope (F3) under a needle sheath, and 14 patients were treated with a Poly optical puncture nephroscope (F4.8). In the direct vision puncture process, the puncture is first completed with B-mode ultrasound localization. The needle tip position is observed by intrathecal endoscopy and the depth of the needle tip in the targeted renal calyx is adjusted properly to ensure that the guide wire in the narrow space of the renal calyx is placed exactly. During the process of localization observation, because of its rigid characteristics, the puncture nephroscope can pass staghorn calculi without hydronephrosis, and is successful regarding the insertion of the guide wire into the renal pelvis and keeping it in the space of UPJ. The puncture needle has a water injection channel to achieve a clear view. Using direct vision, the accuracy of puncture position is determined by the optical puncture technique. When the stone is not observed during the puncture process, the puncture angle can be adjusted. The needle tip position can be observed by intrathecal endoscopy and the depth of needle in the targeted renal calyx can be adjusted properly to ensure that the guide wire in the narrow space of the renal calyx is placed exactly. In addition, B-mode ultrasound localization and optical puncture can be performed simultaneously without increasing the number of punctures. In the 21 patients, the success rate of the first puncture was 95.2% (20/21). Postoperative routine CT examinations of the urinary system can help rule out complications, display residual stones, and help decide whether the nephrostomy tube should be removed. Previous studies suggest that some inconclusive results are to be expected when evaluating a patient using ultrasound, and imaging with a low-dose CT in these circumstances is reasonable [33–35]. Therefore, in the present study, a CT scan was performed 1–3 days after the operation.

Another difficulty in the operation of kidney stones without hydronephrosis is channel establishment. The space of the targeted renal calyx is occupied by stones. After puncture needle enters the targeted renal calyx, the guide wire can easily become curled in the renal calyx or be blocked by the stone in calyceal fornix, and cannot enter the renal pelvis and ureter. In this case, the guide wire can easily slide out of the kidney calyx when the channel is expanded step by step, leading to loss of channel and injury to the renal parenchyma. In order to reduce channel loss, the “two-step method” or the “improved one-step method” is generally used [36, 37]. Although this method can reduce the probability of channel loss to some extent, it still damages the renal parenchyma due to the shearing force of axial expansion. If the puncture needle is not fully perpendicular to the fornix of calix at the time of puncture, it might damage the interlobar artery and cause bleeding during dilation. We selected the method of balloon expansion when we established the channel. In this method, the balloon catheter is pushed along the guide wire under real-time B-mode ultrasound guidance after the guide wire is confirmed to be into the collection system. The puncture channel is expanded by water injection. This is a “coaxial radial one-step dilatation” method for the establishment of skin-kidney channel. It converts the axial shearing force of the traditional fascial dilators into a radial compression force, reducing the cutting damage to the renal tissue and performing some kind of compression hemostasis when expanding.

A number of studies demonstrated that balloon dilation has more advantages than traditional fascial dilators in establishing channels, especially in patients with kidney stone without hydronephrosis [13, 23, 38]. Balloon dilation can significantly increase the success rate of first dilation and reduce the surgical risks. On the other hand, some studies suggested that there is no significant difference in the effects of balloon dilators and Amplaz fascial dilators and metal sleeve dilators during PCNL surgery [11, 12]. It is worth noting that these negative studies were not performed in patients with kidney stones without hydronephrosis.

In our study, PCNL was performed in 21 patients with kidney stone without hydronephrosis, of which 17 patients underwent tubeless treatment without retention of nephrostomy tube, and 5 patients underwent completely tubeless treatment. None of the 17 cases had delayed hemorrhage, sepsis, or perirenal effusion/hematoma after surgery. In a previous study [39], we summarized the surgical experience of tubeless PCNL with common puncture dilation standard channel. The criteria for the selection of tubeless treatment are: 1) no active bleeding or hemorrhage spot, or the hemorrhage of small blood vessels was stopped by bipolar columnar electrocoagulation rod/Nd laser; 2) no injury to the collecting system; 3) no severe hydronephrosis and intrarenal infection; 4) no obvious residual stone; and 5) 11 intercostals or 12 subcostal single channel. We hypothesize that optical puncture combined with balloon dilation standard-channel PCNL lithotripsy can increase the accuracy of puncture and reduce the skin-kidney channel loss, leading to smaller rates of repeated puncture. Thus, it can decrease blood loss and increase the tubeless rate, which may relieve postoperative pain. It can also reduce the length of hospital stay. Nevertheless, we should pay attention on the additional expense of this technique. Both the additionally needed scope and balloon add to the total cost.

This study has limitations. It was not a prospective study and there was probably an inherent selection bias. The sample size was small. There were some subjective judgments on the determination of tubeless treatment selection. The effectiveness still needs to be confirmed by multicenter, large-sample studies.

## Conclusions

Our study suggests that optical puncture combined with balloon dilatation standard channel PCNL lithotripsy in patients with kidney stone > 2 cm without hydronephrosis can increase the accuracy of puncture and success rate of the skin-kidney channel establishment, ensure the stone removal rate, and increase the postoperative tubeless rate. As a result, it can also reduce the length of hospital stay and postoperative pain, and promote rapid recovery after PCNL.

## Data Availability

The datasets used and/or analyzed during the current study are available from the corresponding author on reasonable request.

## Abbreviations

PCNL: percutaneous nephrolithotomy
VAS: visual analogue scale
